# A Facile and Green Synthesis of a MoO_2_-Reduced Graphene Oxide Aerogel for Energy Storage Devices

**DOI:** 10.3390/ma13030594

**Published:** 2020-01-28

**Authors:** Mara Serrapede, Marco Fontana, Arnaud Gigot, Marco Armandi, Glenda Biasotto, Elena Tresso, Paola Rivolo

**Affiliations:** 1 Center for Sustainable Future Technologies, Istituto Italiano di Tecnologia, Via Livorno 60, I-10144 Torino, Italy; mara.serrapede@iit.it (M.S.); marco.fontana@iit.it (M.F.); 2 Department of Applied Science and Technology, Politecnico di Torino, C.so Duca degli Abruzzi 24, I-10129 Torino, Italy; gigot.arnaud@gmail.com (A.G.); marco.armandi@polito.it (M.A.); elena.tresso@polito.it (E.T.); 3 Interdisciplinary Laboratory of Electrochemistry and Ceramics (LIEC), Institute of Chemistry, São Paulo State University-UNESP, Araraquara, SP 14800-060, Brazil; glendabiasotto@uol.com.br

**Keywords:** *l*-ascorbic acid, reduced graphene oxide, aerogels, molybdenum oxide, supercapacitors, electrochemical impedance spectroscopy

## Abstract

A simple, low cost, and “green” method of hydrothermal synthesis, based on the addition of *l*-ascorbic acid (*l*-AA) as a reducing agent, is presented in order to obtain reduced graphene oxide (rGO) and hybrid rGO-MoO_2_ aerogels for the fabrication of supercapacitors. The resulting high degree of chemical reduction of graphene oxide (GO), confirmed by X-Ray Photoelectron Spectroscopy (XPS) analysis, is shown to produce a better electrical double layer (EDL) capacitance, as shown by cyclic voltammetric (CV) measurements. Moreover, a good reduction yield of the carbonaceous 3D-scaffold seems to be achievable even when the precursor of molybdenum oxide is added to the pristine slurry in order to get the hybrid rGO-MoO_2_ compound. The pseudocapacitance contribution from the resulting embedded MoO_2_ microstructures, was then studied by means of CV and electrochemical impedance spectroscopy (EIS). The oxidation state of the molybdenum in the MoO_2_ particles embedded in the rGO aerogel was deeply studied by means of XPS analysis and valuable information on the electrochemical behavior, according to the involved redox reactions, was obtained. Finally, the increased stability of the aerogels prepared with *l*-AA, after charge-discharge cycling, was demonstrated and confirmed by means of Field Emission Scanning Electron Microscopy (FESEM) characterization.

## 1. Introduction

One of the key issues for future energy storage systems is to find new materials for highly efficient electrodes to be exploited in supercapacitors (SCs). Reduced graphene oxide (rGO) has gained, in the last years, great attention due to the following advantages:

- It is produced by using inexpensive graphite as raw material through cost-effective chemical methods with a high yield [1];
- It is highly hydrophilic and it can form stable aqueous colloids to facilitate the assembly of macroscopic structures, when subjected to specific procedures [2].

Any reduction protocol attempts to produce graphene-like materials similar to the pristine graphene, obtained from graphite, both in structure and properties and, in addition, numerous efforts have been made trying to limit the residual functional groups and defects that could alter the structure of the carbon planes.

In addition to the physical techniques, such as thermal annealing [1], microwave [3], photoreduction [4], and hydrothermal processes [5,6], in which water acts as reducing agent, many reducing reactants have been tested. Among them, hydrazine (N_2_H_4_) [7,8,9] sodium bisulfite (NaHSO_3_) [10], sodium borohydride (NaBH_4_) [11], hydriodic acid (HI) [12], sodium iodide (NaI) [13], hypophosphorous acid (H_3_PO_2_) and iodine (I_2_) [14], hydroquinone [10,15], pyrogallol and potassium hydroxide (KOH) [7], demonstrated to reduce GO with different yields. However, due to strong bubbling during the N_2_H_4_ or NaBH_4_-mediated reduction process, the forming hydrogel is broken into pieces [16], thus confirming that the use of these two reagents is less preferable than the ethylendiamine (EDA) usage. Indeed, this synthesis of ultralight chemically converted graphene aerogels, characterized by high compressibility and excellent elasticity, was applied for energy dissipation and vibration damping. NaHSO_3_, on the other hand, was successfully used for preparation of rGO based 3D architecture, but the resulting aerogel showed [10] an electrical conductivity and a C/O ratio lower than the HI mediated reduced material.

Moreover, it was demonstrated that HI produces aerogels with good flexibility and improved tensile strength [17]. In addition, no gaseous products are released with respect to aqueous N_2_H_4_ or NaBH_4_ solutions and thus the integrity and flexibility of the rGOs are not destroyed during the reaction. However, as in the case for aromatic compounds such as pyrogallol and hydroquinone, the reactant is not environmentally friendly.

The usage of green chemistry [18] techniques in material synthesis is highly appealing for numerous reasons, such as environmental sustainability and for cutting down the costs of production and disposal.

Recently [19], phenylalanine was used as a reducing agent to obtain superhydrophobic rGO based aerogels, but, in view of the preparation of electrode pastes to be employed in the presence of aqueous electrolytes, the choice of *l*-ascorbic acid (*l*-AA), seems to be more suitable, also because the oxidized by-product (dehydroascorbic acid) resulting from the reaction is environmentally friendly [20].

A lot of other advantages, provided by *l*-AA, are widely supported by literature, for example, to simply obtain highly reduced suspensions from GO nanosheet in water dispersion or hydrogels at RT or quite mild temperatures, directly in the presence of *l*-AA [7,16,20] or by adding it as a post-process reactant after a GO hydrogel formation [21]. More recently, *l*-AA was added as reducing agent to get very complex hybrid material such as polymers-graphene based aerogels with particular flexibility and mechanical strength properties [22].

As a matter of fact, as reported in previous works [23,24], the hydrothermal synthesis would have to ensure a satisfying degree of GO reduction. However, the addition of *l*-AA to the pristine aqueous dispersion containing the GO flakes would have to increase the yield of reduction directly during the formation of the 3D networked hydrogel, by means of the hydrothermal process. The described procedure is fast and avoids long post-processing steps (such as purification washing). Moreover, in view of the preparation of hybrid aerogels, based on rGO and transition metal compounds, the maximization of the reduction degree, that could be increased by simply varying the amount of reduction agent added to the synthesis batch, allows to enhance the pure Electrical Double Layer (EDL) capacitance, neat of the oxidation-reduction mechanisms involving the oxygen containing species of a partially reduced GO [25], and, hopefully, to provide constant currents over the whole potential window.

Concerning the hybrid aerogels, the *l*-AA was used in the room temperature synthesis of core-shell Cu@Cu_2_O nanoparticles supported on rGO, starting from GO and copper sulfate [26] for catalytic applications and, with an excess of the reducing agent, in the preparation of rGO/Pt nanoparticle hybrids devoted to the transdermal systems for the controlled delivery of vitamin C, tissue engineering, and biosensors [27]. Anodes for Li-ion batteries have been prepared with rGO-MoO_2_ based microspheres, by means of *l*-AA as reducing agent, at a controlled temperature and pressure of 200 °C and 500 Torr, respectively, for 30 min only, and then annealed at 500 °C [28]. More recently, an all-solid-state flexible asymmetric supercapacitor based on the coupling of 3D graphene aerogel with a 3D porous graphene/MnO_2_@polyaniline hybrid film [29] was proposed, where the former only was reduced in autoclave, in a *l*-AA solution, whereas, the latter, under hydrazine vapors. Thus, to the best of authors’ knowledge, the preparation of rGO-MoO_2_ aerogels to be employed in supercapacitors, by means of a one-pot hydrothermal synthesis in the presence of *l*-AA, has not been proposed before. The expected advantages are related to the reduction degree of rGO and to the molybdenum oxidation state (in the transition metal oxide), which can be more controllable, owing to the addition of a *l*-AA suitable amount, thus influencing, in the electrodes for supercapacitors, the material behavior in terms of EDL and pseudocapacitive contributions and stability during cycling [30].

The aim of the work consists in the preparation, morphological, and compositional characterization of rGO and rGO-MoO_2_ aerogels and in the study, by means of cyclic voltammetry (CV) and electrochemical impedance spectroscopy (EIS), of promising electrodes for supercapacitors based on the above mentioned materials, pointing out the added value of *l*-ascorbic acid for the synthesis of electrodes materials which show good properties in terms of stability in symmetric devices.

## 2. Materials and Methods

### 2.1. Preparation of rGO and rGO-MoO_2_ Aerogels

Reduced graphene oxide (rGO) aerogels, containing *l*-ascorbic acid (Sigma-Aldrich), were prepared by dispersing the graphene oxide (GO) powder (Single Layer GO, 0.7−1.2 nm, purchased from Cheap Tubes Inc., Grafton VT, USA) in dH_2_O (volume 17 mL) according to a 2 mg/mL concentration (w/v) and by adding to the slurry different amounts of *l*-AA (1 and 2 mg/mL, respectively), to explore the effect of the reducing agent/GO ratio on the resulting rGO reduction yield. After 5 h, the dispersion was sonicated for 30 min and transferred into a Teflon reactor contained in a stainless-steel autoclave, in order to carry out, in a muffle oven, the hydrothermal reaction, for 12 h at 180 °C. After natural cooling to room temperature, the obtained rGO hydrogel was frozen at –196 °C in liquid nitrogen and then dried, overnight, under vacuum (pressure _in chamber_ ~3 × 10^−3^ mbar) at −55 °C in a LIO—5P DIGITAL lyophilizer (5Pascal-Italy, Milano, Italy). By the same procedure, the rGO without addictive reducing agent (*l*-AA) was prepared for the sake of comparison.

rGO-MoO_2_ hybrid was obtained by adding to the pristine GO slurry (2 mg/mL), after the 25′ sonication, 0.5 g of a phosphomolybdic acid (H_3_PMo_12_O_40_) solution (20 wt% in ethanol, from Sigma-Aldrich) and *l*-AA (2 mg/mL concentration (w/v), for a total volume of 17 mL). The mixture was further sonicated for 5 min to ensure a homogeneous dispersion of the MoO_2_ precursor before the hydrothermal reduction and transferred into a Teflon reactor contained in a stainless-steel autoclave. The hydrothermal reaction to obtain the MoO_2_ decorated rGO hydrogel occurred for 12 h at 180 °C in a muffle oven and, after natural cooling to room temperature, the hydrogel was frozen at −196 °C in liquid nitrogen and then subjected, overnight, to the freeze drying step in the lyophilizer (T = −55 °C, pressure _in chamber_ ~3 × 10^−3^ mbar).

In the following, the aerogels prepared in the presence of *l*-AA will be mentioned as rGO-*vitC* and rGO-MoO_2_-*vitC*.

### 2.2. Materials Characterizations

Specific surface area (SSA) measurements and pore size analysis were carried out on samples previously out-gassed for at least 4 h at 100 °C, to remove water and other atmospheric contaminants, by means of N_2_ isotherms at −196 °C (Quantachrome Autosorb 1C instrument, Boynton Beach, FL, USA). BET SSA values were measured by the multipoint method in the relative pressure range of P/P^0^ = 0.05–0.20; cumulative pore volume curves were obtained by applying the QS-DFT method with appropriate kernel (N_2_ adsorption @ −196 °C onto carbon slit pores).

The morphology of the different aerogels was investigated by means of Field Emission Scanning Electron Microscopy (using a FESEM Supra 40 manufactured by ZEISS, Jena, Germany), equipped with Oxford Si(Li) detector for Energy Dispersive X-Ray Analysis (EDX).

X-Ray Photoelectron Spectroscopy (XPS) was carried out on a PHI 5000 VersaProbe (Physical Electronics-ULVAC-PHI Inc., Chanhassen, MNM USA) system, with a monochromatic Al Kα radiation (1486.6 eV energy) as an X-ray source. Different pass energy values were used for survey (187.75 eV) and HR spectra (23.5 eV). During the measurements, charge compensation was obtained by a combined electron and Ar neutralizer system. The binding energy scale was calibrated by using the main C1s contribution (C–C/C–H bonds, 284.5 eV) as reference value. Concerning the analysis of HR scans, the background contribution was modeled with a Shirley function [31]. CasaXPS software (Casa Software Ltd., Teignmouth, UK) was used for peak devoncolution, semi-quantatitve analysis, and calculation of uncertainties by means of Monte Carlo routines. Concerning the peak fitting procedure, two types of lineshapes were exploited: GL(m) and LF(α, β, w, m).

X-Ray Diffraction Analysis (XRD, Panalytical X’Pert MRD Pro Cu Ka X-ray source, Malvern Panalytical Rtd, Malvern, UK) in Bragg/Brentano configuration was used to assess the structural characteristics of the pure rGO and hybrid aerogels.

### 2.3. Electrodes Preparation

Working electrodes were fabricated by drop-casting a well-sonicated solution made by mixing the as-synthetized active material with a conductive agent and a binder in absolute ethanol [32], as homogenizing solvent, onto a well-polished glassy carbon electrode (diameter of 0.3 cm, BioLogic). Experiments in planar symmetric device configuration were carried out with Fluorine doped Tin Oxide glass (FTO) as current collectors (diameter of 0.5 cm, Solaronix, 10 Ω/sq) onto which the slurry was drop-casted and left to dry in open air.

The slurry had the composition of 8 mg active material, 0.4 mg acetylene black (Alfa Aesar), and 5 µL Nafion^®^ 5% (Sigma-Aldrich) dispersed in water in order to obtain a whole volume of 0.557 mL. Electrodes where therefore obtained by drop-casting the ink in order to load the current collectors with 0.5 mg/cm^2^ of active material. The electrodes were left in air to dry overnight before starting the experiments in analytical electrochemical cell.

In three electrodes experiments, a platinum bar and a homemade saturated mercurous sulfate electrode SMSE (680 mV vs. standard hydrogen electrode, SHE) or a saturated calomel electrode, SCE (240 mV vs. SHE) electrodes were used as counter and reference electrodes, respectively. The comparison between pristine rGO and rGO-*vitC* (2 mg/mL) were performed in 1 M NaCl, while all the other tests were all carried out in 1 M Na_2_SO_4_.

In symmetrical devices, two identical electrodes made with FTO as current collector were faced in planar configuration with a glass-frit membrane as separator (Whatman GF/A) and a melted thin thermoplastic polymer (Parafilm^®^) as sealant.

All the electrochemical measurements were carried out with milliQ water (18.2 MΩ cm^−1^) as solvent after soaking the electrodes in the electrolyte for 24 h.

### 2.4. Electrochemical Characterization

All the measurements were performed on a Metrohm Autolab PGSTATM101 potentiostat-galvanostat. In three electrodes cells, cyclic voltammetry (CV) was carried out at multiple scan rates, galvanostatic charge-discharge cycles at current densities of 85, 170, and 350 mA g^−1^ and AC impedance spectroscopy (EIS) was done at open circuit potential (OCP) in the frequency range from 10 kHz to 1 mHz with 5 mV amplitude. In device configuration, the samples were aged by performing CV for 50,000 cycles at 0.2 V s^−1^ and acquiring a slow voltammogram every 1000 cycles that was analyzed to estimate the capacitance. After the slow voltammogram, AC impedance spectroscopy was acquired at 0 V in the frequency range from 10 kHz to 10 mHz with 5 mV amplitude.

## 3. Results and Discussion

### 3.1. Chemical-Physical Characterization

#### 3.1.1. rGO and rGO-vitC

**Figure 1:**
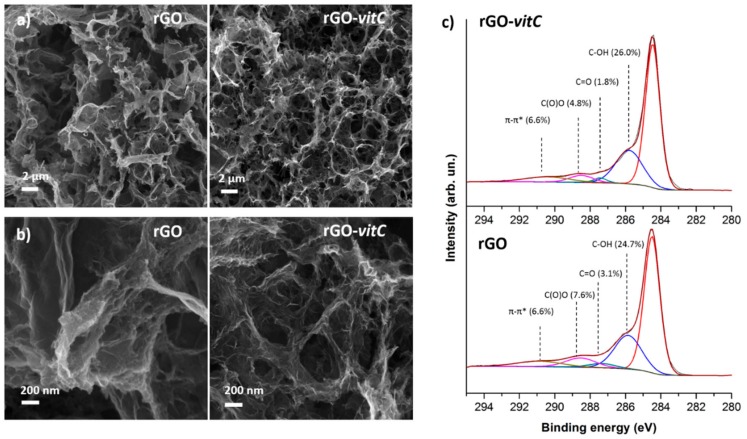
Morphological comparison between reduced graphene oxide (rGO) and rGO-*vitC* aerogels at low (**a**) and high (**b**) magnification by FESEM images; (**c**) reports the deconvolution through synthetic components of the C1s region of the photoelectron spectrum for both materials.

Figure 1a provides a morphological comparison through FESEM images between rGO aerogels synthesized in the absence of the reducing agent and in the presence of the maximum tested *l*-AA concentration (2 mg/mL), where it is evident that both aerogels exhibit the distinctive porous 3D structure of rGO aerogels [5,24] constituted of interconnected, wrinkled flakes. FESEM images at different magnifications show that the morphology in both cases is comparable, as it is characterized by pores whose size ranges from the nanometer to the micrometer scale. On the other hand, at the nanoscale (Figure 1b), extremely wrinkled flakes are obtained by means of both the synthesis routes thus leading to high specific surface area (SSA) and accessibility by a liquid electrolyte, as the hydrothermal synthesis itself partially prevents the graphene sheets restacking.

Since FESEM cannot provide quantitative analysis of pore size and SSA at the nanoscale, the porosity of the aerogels was studied by means of N_2_ isotherms and evaluated through BET theory.

**Figure 2:**
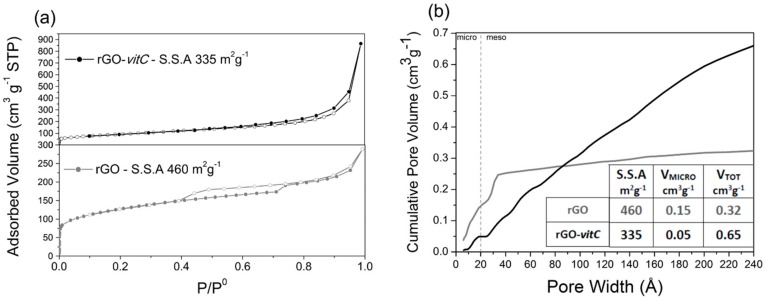
N_2_ adsorption/desorption isotherms at 77 K of (lower) rGO and (upper) rGO-*vitC-*2 mg/mL (**a**); cumulative pore volume curves of rGO (gray) and rGO-*vitC* (black) (**b**).

The N_2_ adsorption/desorption isotherms at 77 K and cumulative pore volume curves of rGO and rGO-*vitC* are reported in Figure 2a,b, respectively. The isotherm of rGO (lower part) shows an adsorption branch composed of Type I(b) (low P/P^0^ region, associated with micropores filling) and Type II (high P/P^0^ region, associated with mesopores filling) [33]. H4 Type hysteresis loop, with the lower limit of the desorption branch located at the cavitation-induced P/P^0^ (i.e., ≈ 0.4) is observed, indicating the presence of mesopores accessible to the outer surface only through narrower necks. BET SSA of 460 m^2^g^−1^ was calculated for rGO. On the other hand, a Type II isotherm with limited hysteresis loop is observed with rGO-*vitC* (2 mg/mL), resulting in a lower SSA (335 m^2^g^−1^).

According to the different isotherm shapes, the cumulative pore volume curves of the two samples display different profiles. Whereas the SSA of rGO is clearly due to micropores and narrow mesopores (i.e., pore width smaller than 3 nm), likely formed by partially (disorderly) stacked graphene sheets, the one of rGO-*vit-C* is mainly due to mesopores with a broad distribution of width, ranging from 2 to more than 20 nm. The presence of *l*-AA in the slurry, subjected to the hydrothermal process, seems to induce the formation a more open structure, likely due to the evolution of small gas bubbles (e.g., H_2_) during the reduction process.

XPS analysis is commonly adopted for the evaluation of reduction of GO by means of semi-quantitative analysis of spectra which provide C/O atomic concentration ratio [1] and deconvolution of the C1s region of the photoelectron spectrum provides further insight into residual oxygen functionalities.

At first, survey spectra were acquired (see Figure S1 in Supplementary Materials), highlighting the presence of C and O chemical elements and therefore confirming the absence of any contamination at concentrations over the detection limit (≈ 0.1% At). Semiquantitative results (Table 1) were obtained by integrating peak areas under the C1s and O1s regions of the photoelectron spectrum, using tabulated relative sensitivity factors (RSF) specified by the manufacturer of the instrument for the analysis. It is interesting to notice that *l*-AA leads to more reduced rGO, according to an upward trend, as validated by the C/O atomic concentration ratio (≈ 7.5). This result comes from the rGO obtained with the highest concentration of *l*-AA tested, which is higher than the one (≈6.3) related to the rGO prepared with a medium concentration of *l*-AA, which, in turn, is higher than the one of the pristine rGO (≈ 5.4): The three types of rGO have a C/O ratio significantly higher than the starting GO (≈ 1.9). The C1s region (Figure 1c) of the photoelectron spectrum suggests that the higher C/O ratio in rGO-*vitC*-2 mg/mL can be attributed to the highly reduced concentration of C=O and C(O)O groups, which compensates the slight increase in C-OH groups.

These values derive from several overlapping of synthetic components, thus uncertainties must be taken into account, as explained in the supporting information (Figure S2).

#### 3.1.2. rGO-MoO_2_-*vit*C

The hybrid rGO-*vitC* aerogel decorated with MoO_2_ microparticles was synthesized by adding phosphomolybdic acid (H_3_PMo_12_O_40_) to the pristine aqueous solution, in the presence of the maximum tested concentration of *l*-AA (2 mg/mL), only (as described in Paragraph 2.1), since it could fairly ensure an high degree of chemical reduction also for the molybdenum in the resulting oxide (from Mo (VI) to Mo (IV)).

**Figure 3:**
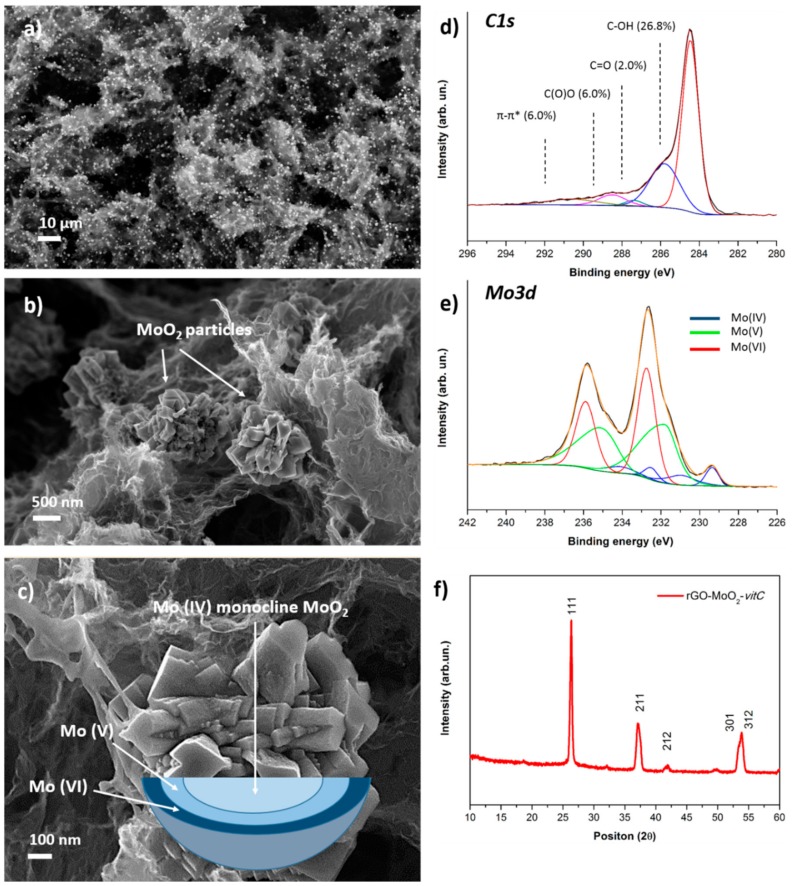
FESEM images at low (**a**) and high (**b**) magnification of the rGO-MoO_2_-*vitC* sample; (**c**) FESEM image of a MoO_2_ particle with a schematic representation of the gradient of Mo oxidation state. XPS high-resolution scans of C1s (**d**) and Mo3d (**e**) regions for rGO-MoO_2_-*vitC* sample; (**f**) provides the XRD spectrum of the hybrid aerogel.

The morphology of the obtained aerogel was characterized by FESEM, as shown in Figure 3a–c. The distinctive quality of the hybrid material is the presence of micrometric particles with nanostructured surface which are embedded in the characteristic 3D structure of the aerogel. It is interesting to observe a homogeneous distribution of the particles in the rGO matrix, as clearly demonstrated by low-magnification images such as Figure 3a, where the particles are noticeable as higher-intensity features. Figure 3f reports the XRD pattern of the hybrid rGO-MoO_2_-*vitC* aerogel, which provides evidence of the formation of MoO_2_ monocline phase, in accordance with JCPDS card No. 32-0671.

XPS was performed to verify the successful reduction of the starting GO in the presence of the Mo precursor and to investigate the chemical composition at the surface of the microparticles. Analysis of the C1s region (Figure 3d) of the photoelectron spectrum provides evidence of the reduction of GO, as it shows comparable peak structure to rGO and rGO-*vitC* aerogels, whereas the Mo3d region (Figure 3e) is rich in information, concerning the identification of molybdenum-containing phases, although it requires great care in the peak-fitting procedure.

The set of four peaks at lower binding energy was assigned to Mo (IV); specifically, the peaks at 229.4 and 232.5 eV are ascribed to the usual MoO_2_ spin-orbit doublet, while the satellite peaks at 230.9 and 234.1 eV are associated to unscreened final states, as theoretically predicted and experimentally observed for crystalline MoO_2_ [34].

The broad asymmetric peaks at 231.8 and 234.6 eV are interpreted as Mo (V) species, although we have slightly lower peak splitting than the usual separation of spin-orbit components in molybdenum (2.8 eV instead of ≈ 3.1 eV). However, we highlight that the interpretation of Mo (V) components is not straight-forward and it is a subject of debate in the literature, so we based our analysis on a recently published work [35], where the peak deconvolution of the Mo3d region for mixed-oxide states was achieved by means of a multivariate approach. The interpretation of the doublet at higher binding energy (232.7, 235.9 eV) is simpler, with binding energy values characteristic of MoO_3_ (Mo (VI) oxidation state) according to the literature [36]. In summary, XPS highlights the co-presence of mixed oxide phases (specifically MoO_2_, Mo_2_O_3_, and MoO_3_), a phenomenon already reported in the literature [24,37]. Moreover, since XPS sampling depth is < 10 nm and conventional XRD is a bulk-analysis technique, we suppose that the microparticles are composed by a monocline MoO_2_ phase surrounded by mixed molybdenum oxide phases, at the near surface, following a gradient of molybdenum oxidation states from IV (core) to VI (surface), according to the oxidation effect of air oxygen, as depicted in the scheme of Figure 3c.

### 3.2. Electrochemical Characterization

In this section, the pure rGO obtained without any addition of reducing agents and the rGO (hybrid and not) prepared with *l*-AA at 2 mg/mL of w/v concentration will be considered only.

#### 3.2.1. Comparison between rGO obtained without and with *l*-AA

The addition of *l*-AA (2 mg/mL) to the reactants to be used in the hydrothermal synthesis of rGO leads to a material with electrochemical performances quite different from those obtained with an identical procedure without the addition of any reducing agent. From the experiments acquired in 1 M NaCl it is clear that the two samples have fairly different potential windows (defined on a coulombic efficiency of the 95% at 5 mVs^−1^).

**Figure 4:**
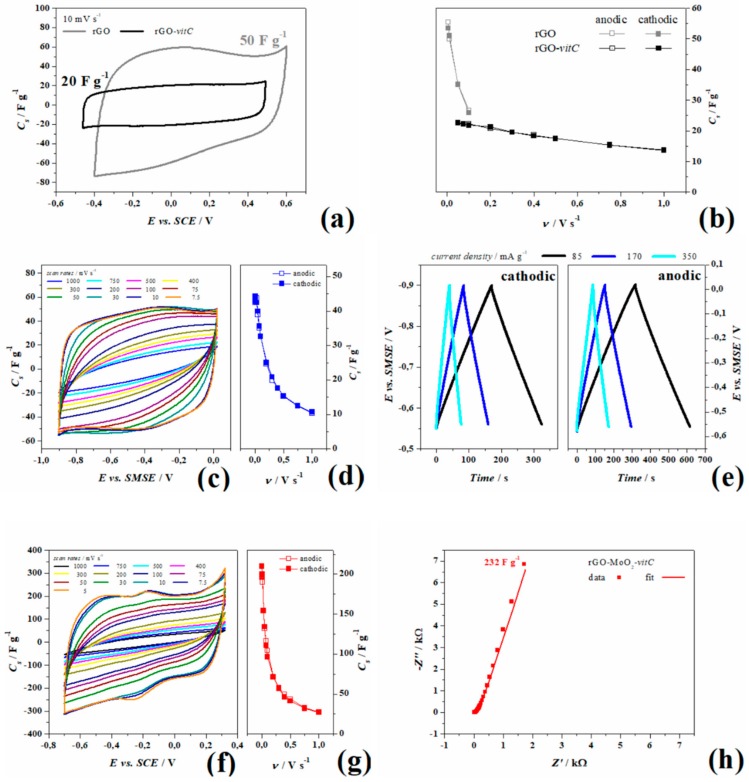
Comparison in 1 M NaCl of pristine rGO (gray) and rGO-*vitC* (black) in (**a**) cyclic voltammetry at 10 mV s^−1^ and (**b**) specific capacitance at multiple scan rates. Experiments carried out 1 M Na_2_SO_4_: (**c**) Cyclic voltammetry, (**d**) specific capacitance at multiple scan rates, and (**e**) galvanostatic charge/discharge cycles of rGO-*vitC*; (**f**) cyclic voltammetry, (**g**) specific capacitance at multiple scan rates, and (**h**) Nyquist plot at open circuit potential (OCP) of rGO-MoO_2_-vitC.

In fact, from Figure 4a, the box-like voltammogram in cyclic voltammetry which is the characteristic of purely electrical double layer interfaces, is measured for the rGO-*vitC* only, while the pristine rGO shows bumps and asymmetry, ascribable to –C=O, –C(O)O, –C–OH bonds. The almost perfect rectangular shape obtained in the rGO-*vitC* is due to a higher yield of reduction of the GO flakes, as previously shown from XPS analysis in Table 1, even if the much smaller area underlying the voltammogram indicates less storage capability of the rGO-*vitC* (at 10 mV s^−1^). The same behavior was observed at multiple scan rates, as shown in Figure 4b, with a maximum capacitance of 55 and 23 F g^−1^ in rGO and rGO-*vitC*, respectively. This trend is likely due to the lower active surface area and to the broader pore size distribution (mainly consisting of mesopores) observed with rGO-*vitC* (see Section 3.1.1).

Moreover, AC impedance experiments, performed at OCP and shown in Figure S3, are consistent with the voltammetric behavior showing better performances as purely EDL capacitor for rGO-*vitC*, since it provides a larger maximum phase in the low frequency domain (82/90°) and a smaller time constant (τ_0_ = 0.6 s) than those measured for the pristine rGO (78/90° and 22.5 s, respectively). Akin the smaller area under the voltammograms, the impedance module at 3 mHz of pristine rGO is 2.5 and 15 kΩ for rGO-*vitC*.

#### 3.2.2. Effect of *l*-Ascorbic Acid on rGO-vitC and rGO-MoO_2_-vitC: Three electrode and device performances

Experiments in 1 M Na_2_SO_4_ were carried out for rGO and rGO-MoO_2_ both synthetized in the presence of *l*-AA (2 mg/mL) in three electrode cells and in planar symmetrical device to study the cyclability during the ageing procedure. Figure 4c,d show the cyclic voltammetry of rGO-*vitC* recorded at various scan rates ranging from 1 V s^−1^ to 7.5 mV s^−1^ and the corresponding specific capacitance (values are summarized in Table 2), respectively. In Figure 4e, instead, galvanostatic charge-discharge experiments acquired at three current densities show the linear behavior of the potential from OCP (−0.55 V vs. SMSE) to the lower potential limit (Figure 4e left-hand side, obtained by applying negative currents) and from OCP to the higher potential limit (Figure 4e right-hand side, obtained by applying positive currents), as well as, the chronopotentiograms are all perfectly linear in the complete potential ranges (cathodic to OCP and anodic to OCP) and show little ohmic drops (8 Ω). Both the experiments consistently measure a maximum specific capacitance of 44.5 F g^−1^ from anodic galvanostatic discharge and 44 F g^−1^ from voltammetry during the anodic sweep.

rGO-MoO_2_-*vitC* was tested likewise and Figure 4f, g show the CV with the specific capacitance estimated per each scan rate (values are reported in Table 2). It is worth noticing that the current related to rGO-MoO_2_-*vitC* increases with respect to rGO-*vitC* only, by reaching the maximum capacitance of 210 F g^−1^ at 5 mV s^−1,^ and that the shape of curves is not rectangular, with a redox reaction at about –0.2 V, observable as the anodic peak at –0.17 V and the cathodic peak at –0.27 V at 5 mVs^−1^. By increasing the sweep rate, the curve shrinks and the peaks gradually move out of the employed potential window, suggesting that, at fast scan rates, the capacitance of rGO-*vitC* and rGO-MoO_2_-*vitC* are quite similar, while, at slow rates, the capacitance of the hybrid rGO increases of one order of magnitude, so indicating the major contribution of the redox processes to the total charge. The potential window chosen in this experiment is quite wide if compared to the thermodynamic database (E° vs. pH) of MoO_2_ in a water-based electrolyte [38]. According to Deltombe et al., in [38], MoO_3_ is stable from pH 0 (E > 0.6 vs. SCE) up to pH 4 (E > 0.3 V vs. SCE) and MoO_2_ can exist in a limited potential window in the whole pH scale and it reaches its maximum potential range (0.4 V) between 2 < pH < 5, while the thermodynamic diagram shows stable MoO_2_ from −0.25 to 0 V vs. SCE at pH 7. For potential lower than −0.25 V, the complete reduction (4e^−^) leads to metallic Mo, whereas for potential higher than 0 V, the solid oxide dissolves in MoO_4_^2−^ with the oxidation to Mo (VI). As previously mentioned for rGO-MoO_2_-*vitC*, the higher intensity of Mo (VI) photoelectrons (Figure 3e) detected onto the MoO_2_ structures suggests a different chemistry in the outer “shell” of the MoO_2_ spheres with Mo multivalent state composition (Figure 3f).

The mechanisms giving rise to the pseudocapacitive behavior of rGO-MoO_2_-*vitC* has been studied on the basis of the potential of the peaks together with the surface Mo valence investigated by XPS before the electrochemical tests. Electrochemical experiments, carried out in previous tests to screen the more suitable potential limits, showed that the as-synthetized aerogel of rGO-MoO_2_-*vitC* undergoes irreversible reactions at E > 0.32 and E< −0.8 V vs. SCE, and a steep increase of the current is recorded with unstable following CV, when the potential exceeds the positive limit of Figure 4f. Moreover, the AC impedance spectra shows a 45° phase in the Bode plot and a semicircle in the Nyquist plot at medium frequencies (Figure S4a–c), indicating diffusion to the electrode followed by a charge transfer that, in the authors’ opinion, is linked to the diffusion of MoO_4_^2−^ species. When the bias is swept to a potential more negative than −0.8 V, a large cathodic asymmetric peak appears in the first cycle, followed by the complete depletion of the anodic and cathodic peaks in the subsequent scan, along with the voltammetry stabilization to a rectangular shape (see Figure S5a).

The electrochemical window employed in the experiments of rGO-MoO_2_-*vitC* was evaluated in order to optimize the redox reversibility for enhanced capacitance and cyclability. In the first cycle, three small peaks rise up from the rectangular voltammetry whose intensity gradually change until peak I and III disappear after 100 cycles and stable voltammograms can be recorded (Figure 4f), owing to the assumption, in accordance with XPS results, that the 10 nm depth “shell” of the spheres, mainly composed of MoO_3−x_ experiences a progressive reduction at the solid state.

When steady voltammograms are recorded, the whole MoO_3-x_ surface with Mo (VI) and Mo (V) has been completely reduced to a stable Mo (IV)O_2_ at neutral pH, as previously reported by Liu et al. [37]. The half-potential of the remaining pair of peaks (II) that do not disappear after cycling is at about −0.2 V vs. SCE, which is the thermodynamic potential of the four electrodes reaction [38]:

$$\mathrm{Mo}+2H_{2}O⥂{\mathrm{MoO}}_{2}+4H^{+}+4e^{-}$$

$$E_{0}=-0.072-0.0591\;\mathrm{pH}$$

The upper limit in cyclic voltammetry of 0.32 V is higher when compared to the value reported in the E_0_-pH diagram at pH = 7, but no dissolution is observed until this potential was reached. Moreover, a stable cyclic voltammetry was recorded as the AC impedance spectrum at OCP measured after cyclic voltammetry experiments shows no 45° phase in the Bode plot nor semicircles followed by diffusion line in the Nyquist plot (Figure S6 and Figure 4h, respectively).

By comparing the rGO-MoO_2_-*vitC* behavior at neutral pH with the one in pH = 2 buffered electrolyte, it is possible to observe that the three peaks are preserved also after 1000 cycles (Figure S5b), no dissolution is monitored and AC impedance spectrum shows features at OCP similar to those obtained in Na_2_SO_4_, such as identical maximum phase of 77° at 1 mHz and time constant of 32 s in acid and 52 s in neutral electrolyte (reported in Figure S6). Therefore, it is reliable that the surface stoichiometry of MoO_3−x_ is maintained with a main core of MoO_2_.

**Figure 5:**
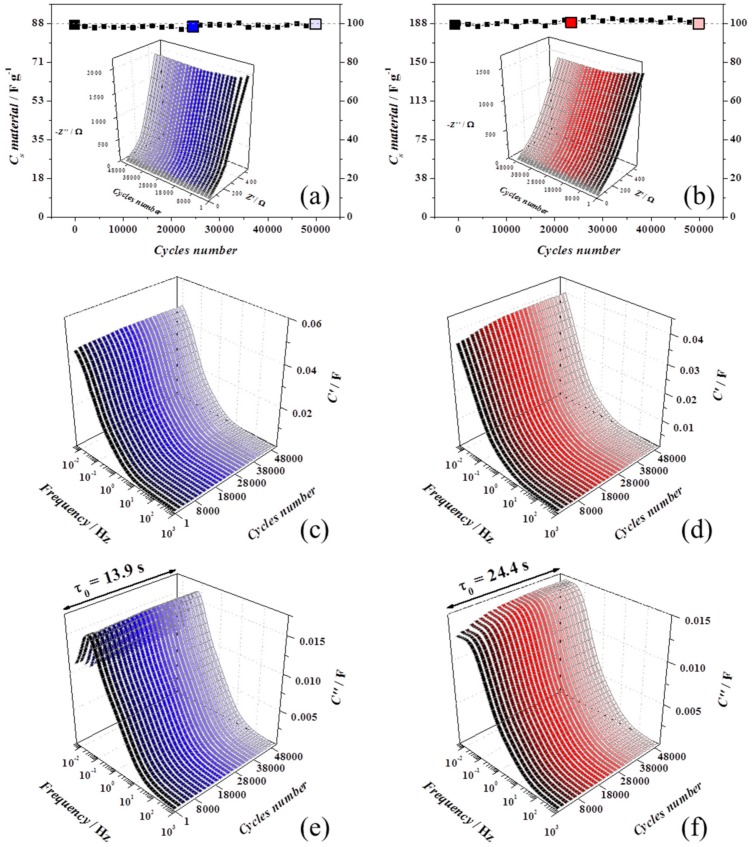
All the ageing experiments were carried out in device with 1 M Na_2_SO_4_. rGO-*vit*C experiments are reported on the left-hand side (blue shadows) and rGO-MoO_2_-vitC experiments are presented on the right-hand side (red shadows). AC impedance spectra were carried out at 0 V every 1000 cycles. (**a**,**b**) Capacitance retention and Nyquist plots in the inset, (**c**,**d**) real part of the complex capacitance spectra (C’), and (**e**,**f**) imaginary part of the complex capacitance spectra (C’’) with the relaxation time constant of the device.

The cycling stability was examined by cycling at 0.2 V s^−1^ for 50,000 cycles, and as shown in Figure 5a,b, both the devices retained 100% of capacitance, showing excellent stability. Every 1000 cycles, a slow voltammogram was recorded to estimate the capacitance and an AC impedance spectrum was measured to monitor if a variation of the equivalent circuit model could be linked to structural changes.

Some differences in the Nyquist (insets in Figure 5a,b) and complex capacitance plots (Figure 5c,d for C’ and Figure 5e,f for C’’) [39] are clear in the first 3000 cycles for rGO-*vitC* (blue graphs, left-hand side) and 8000 cycles for rGO-MoO_2_-*vitC* (red graphs, right-hand side), may be due to activation processes or to progress wettability at the interface which participate to increase the utilized surface. In both, the devices during the just mentioned first cycles, both C’ and C’’ increase towards higher values in the low frequency domain, but together the uncompensated resistance (*R_u_*), the relaxation time constant (*τ_0_*), and the overall capacitance were constant until the end of ageing, meaning that the charge/discharge capabilities are nicely maintained.

**Figure 6:**
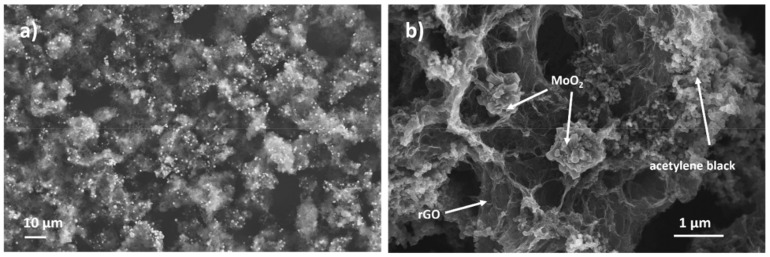
FESEM images of the electrode material containing rGO-MoO_2_-*vitC*, acetylene black, and Nafion after 5000 cycles.

The morphology of the aged rGO-MoO_2_-*vitC* electrode was examined by FESEM-EDS after 50,000 cycles (Figure 6) and there is no evidence of changes of the MoO_2_ structures, confirming the reliability of the analyzed rGO-MoO_2_-*vitC* aerogels.

## 4. Conclusions

*l*-AA, used as a reducing agent in the hydrothermal synthesis, can be used to control the degree of chemical reduction of rGO aerogels by simply varying the amount of added reducing agent, according to an upward trend, as shown by XPS analysis.

Moreover, electrochemical tests have demonstrated that, in pure rGO, a high degree of reduction has been obtained at the maximum *l*-AA concentration used in the synthesis process and, furthermore, a noticeable capacitance retention is achievable, in neutral pH conditions, compatible with green chemistry approach, simple fabrication and safe handling, without structural degradation features, as shown by FESEM micrographs, acquired on the aged devices.

Actually, the oxygen containing functional groups which, in principle, can give more capacitance in pure rGO (obtained without *l*-AA), do not allow to have a relevant stability of the material in water. The rGO, synthesized in the presence of *l*-AA, being less functionalized, even if it shows a lower capacitance, is able to provide constant currents over the whole potential window of the material and thus it is a better material to store charges.

In addition, *l*-AA induces a high degree of reduction not only for the carbonaceous scaffold but also for the Mo cation in the hybrid rGO-MoO_2_ aerogel, as supported by XPS and XRD data. The synthesized aerogels can be easily processed as slurry and employed as active material to fill unusual electrodes layouts for supercapacitors [40].

Indeed, this mild and green synthesis method can be supposed to be a standard procedure for the production of hybrid graphene/transition metal compounds for several applications, such as energy storage devices.

## Figures and Tables

**Table 1:** Atomic concentration values for C and O elements as derived from semiquantitative analysis of XPS spectra.

Sample	C (at%)	O (at%)	C/O
commercial GO	65.3 ± 0.4 *	33.4 ± 0.3 *	1.95 ± 0.02
rGO	84.3 ± 0.3	15.7 ± 0.3	5.4 ± 0.1
rGO-*vitC* (1 mg/mL)	86.3 ± 0.3	13.7 ± 0.3	6.3± 0.1
rGO-*vitC* (2 mg/mL)	88.3 ± 0.3	11.7 ± 0.3	7.5 ± 0.2

* Commercial GO atomic concentration values do not add up to 100% due to 1.3% At of N as residual contamination from the oxidation process of graphite in commercial GO.

**Table 2:** Capacitance estimated during the anodic and cathodic scans at different scan rates recorded on rGO-*vitC* and rGO-MoO_2_-*vitC.*

rGO-*vitC*			rGO-MoO_2_-*vitC*		
Scan Rate	Anodic Scan	Cathodic Scan	Scan Rate	Anodic Scan	Cathodic Scan
(V/s)	(F g^−1^)	(F g^−1^)	(V/s)	(F g^−1^)	(F g^−1^)
1	10	11	1	27	27
0.75	12	12	0.75	33	32
0.5	15	15	0.5	43	41
0.4	17	18	0.4	49	46
0.3	20	21	0.3	58	57
0.2	24	25	0.2	71	71
0.1	33	33	0.1	104	96
0.075	35	36	0.075	117	111
0.05	39	40	0.05	132	134
0.03	44	42	0.03	155	154
0.01	44	44	0.01	190	197
0.0075	44	43	0.0075	196	200
0.005	42	43	0.005	199	210

## Supplementary Materials

The following are available online at https://www.mdpi.com/1996-1944/13/3/594/s1. Supporting Information Serrapede et al. Figure S1. XPS survey spectra for commercial GO, rGO aerogel, rGO-*vitC* (1 mg/mL), and rGO-*vitC* (2 mg/mL) aerogels, Figure S2. XPS C1s peak fitting for rGO, rGO-*vitC* (1 mg/mL), rGO-*vitC* (2 mg/mL), rGO-MoO2-*vitC* aerogels, Figure S3. Comparison in 1 M NaCl of pristine rGO (gray) and rGO-*vitC* (black). From the bottom graph to the top: Bode plot of the modules, Bode plot of the phases, capacitance over maximum capacitance (*C C_0_^−1^* vs. *Frequency*) per each sample, real complex capacitance over maximum real complex capacitance (*C’ C’_0_^−1^* vs. *Frequency*) per each sample, imaginary complex capacitance over maximum imaginary complex capacitance (*C’’ C’’_0_^−1^* vs. *Frequency*) with relaxation time constants per each sample, Figure S4. Experiments carried out 1 M Na_2_SO_4_: (a) Cyclic voltammetry up to 0.4 V, (b) Bode plot of the phase, and (c) Nyquist plot acquired at OCP after 100 cyclic voltammograms at 5 mV s^−1^ in the potential window of experiment (a), Figure S5. Differential voltammetry carried out in (a) 1 M Na_2_SO_4_ with the lower potential limit of −0.9 V and (b) in 1 M PB at pH = 2, Figure S6. Comparison of rGO-MoO_2_*-vitC* in 1 M Na_2_SO_4_ (red) and 1 M PB at pH = 2 (wine). From the bottom graph to the top: Bode plot of the modules, Bode plot of the phases, capacitance over maximum capacitance (*C C_0_^−1^* vs. *Frequency*) per each sample, real complex capacitance over maximum real complex capacitance (*C’ C’_0_^−1^* vs. *Frequency*) per each sample, imaginary complex capacitance over maximum imaginary complex capacitance (*C’’ C’’_0_^−1^* vs. *Frequency*) with relaxation time constants per each sample, Figure S7. EDX spectrum of sample rGO-MoO2 with related semi-quantitative analysis.
