# Supplementary material for: A Facile and Green Synthesis of a MoO2-Reduced Graphene Oxide Aerogel for Energy Storage Devices
Source: Materials (Basel). 2020 Jan 28;13(3):594. doi: 10.3390/ma13030594 (PMC7040781; doi:10.3390/ma13030594)
Supplement: Supplementary file 1 [file materials-13-00594-s001.pdf]

materials

Supporting Information

A Facile and Green Synthesis of a MoO_2-Reduced Graphene Oxide Aerogel for Energy Storage Devices

1. XPS

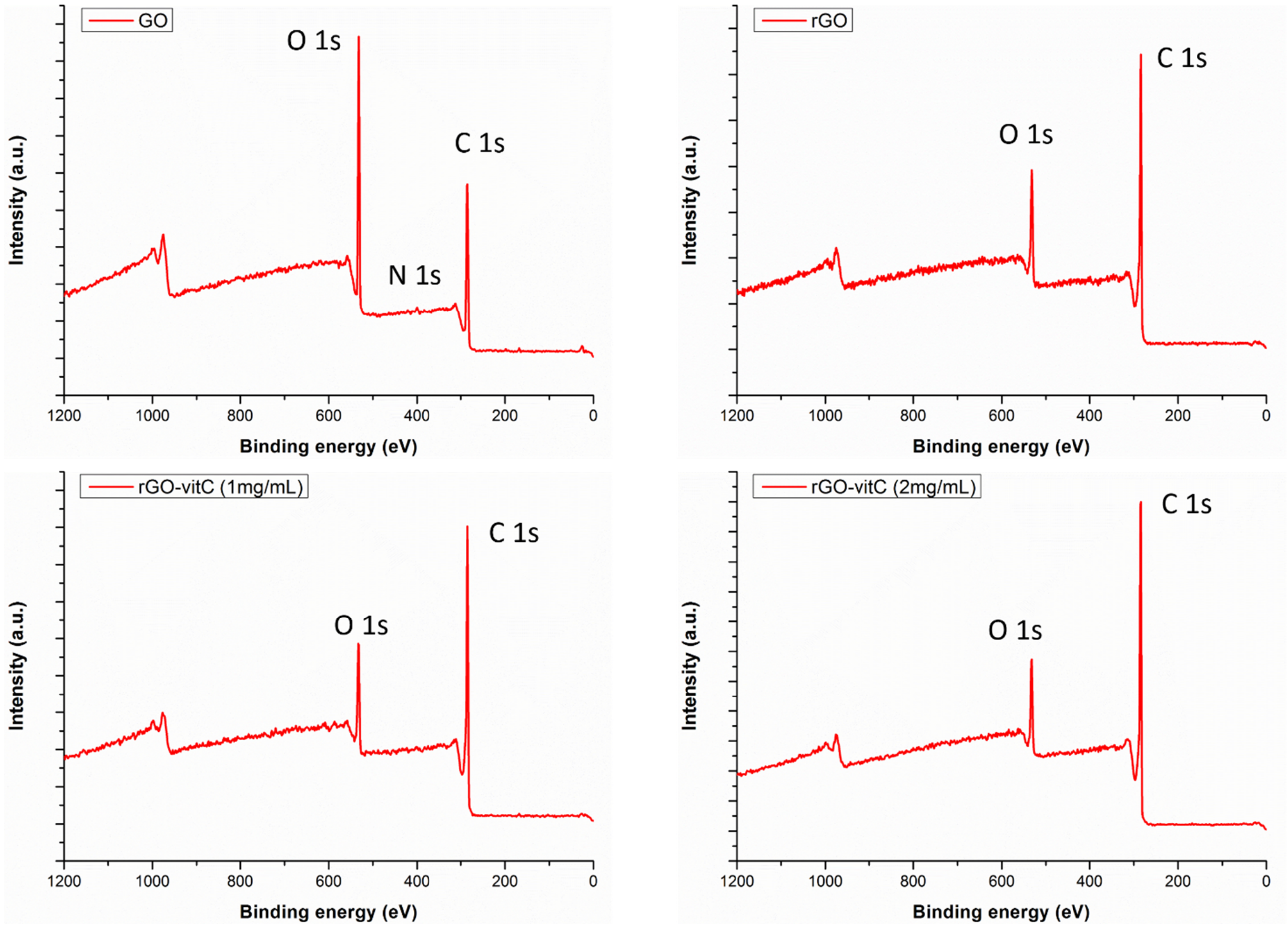

Figure S1. XPS survey spectra for commercial GO, rGO aerogel, rGO-vitC (1 mg/mL) and rGO-vitC (2 mg/mL) aerogels.

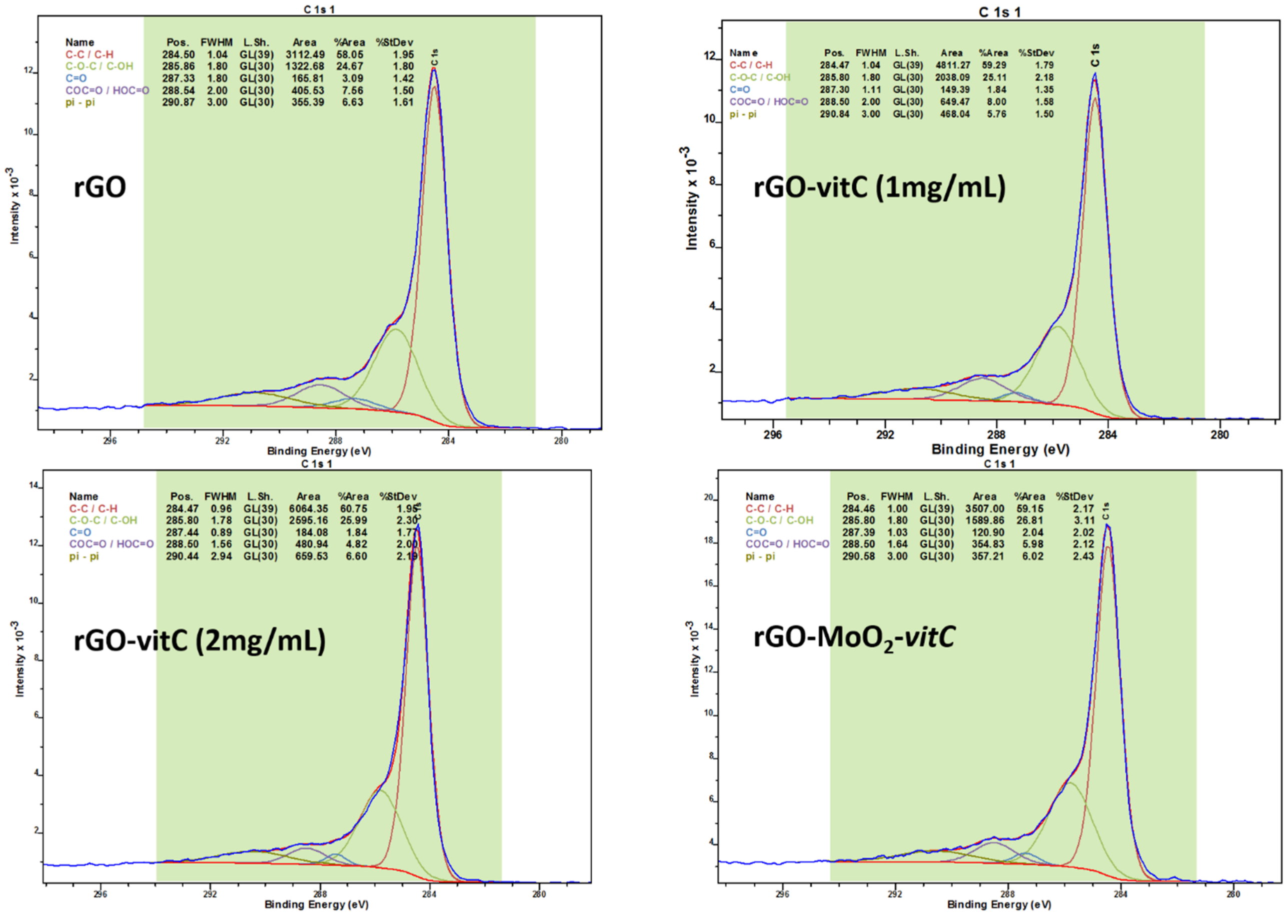

Figure S2. XPS C1s peak fitting for rGO, rGO-vitC (1 mg/mL), rGO-vitC (2 mg/mL), rGO-MoO_2-*vitC* aerogels.

Figure S2 reports the peak deconvolution process for the C1s region for rGO, rGO-*vitC*, rGO-MoO_2-*vitC* with uncertainty values for peak area percentage. It is important to notice that the uncertainties are significant, due to the fact that the synthetic components overlap and have influence on each other.

The reported uncertainties were calculated with CasaXPS, using Monte Carlo routines.

2. Electrochemical Characterization

2.1. Electrodes preparation, electrochemical characterization, and analysis

The working electrodes fabrication and the electrochemical characterization setup is reported in the main article. In addition, herein we add:

- In Figure S3: AC impedance spectra acquired at OCP in 1 M NaCl to compare the electrochemical behavior of rGO and rGO-*vitC*;
- In Figure S4: Cyclic voltammetry at 5 mV s^{-1} and Bode plot of AC impedance measurements acquired on rGO-MoO_2-*vitC* in 1 M Na_2SO_4 to study the electrochemical potential window at E > +0.32 V vs. SCE;
- In Figure S5: The comparison of cyclic voltammograms acquired in 1 M Na_2SO_4 and 1 M phosphate buffer (PB) at pH = 2 (both vs. Ag/AgCl KCl saturated) to study the potential window in neutral electrolyte at E < -0.7 V and to compare the stability of the peaks in neutral and acidic electrolyte;
- In Figure S6: AC impedance spectra of rGO-MoO_2-*vitC* acquired at OCP in 1 M Na_2SO_4 (vs. SCE) and 1 M phosphate buffer (PB) at pH = 2 (vs. Ag/AgCl KCl saturated) to compare the stability of the peaks in neutral and acidic electrolyte.

All the observations of these Figures are reported in the main article.

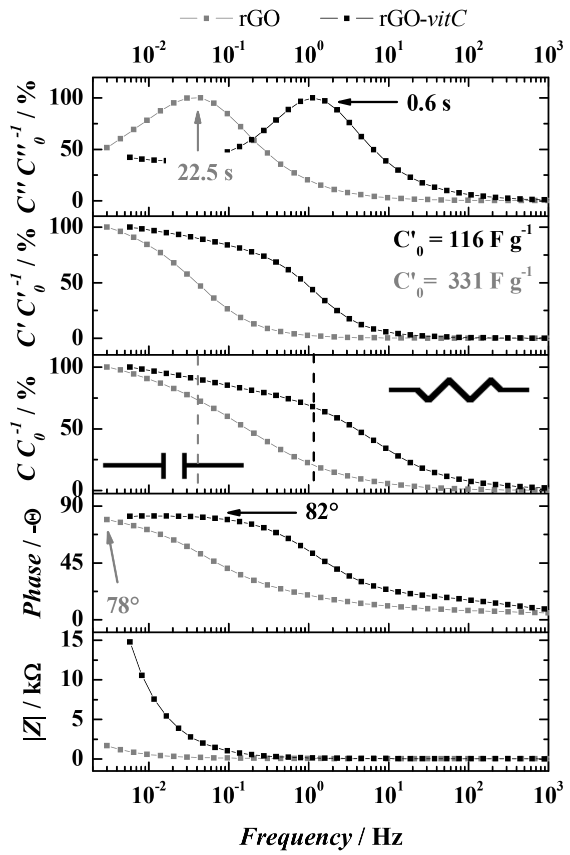

Figure S3. Comparison in 1 M NaCl of pristine rGO (gray) and rGO-*vitC* (black). From the bottom graph to the top: Bode plot of the modules, Bode plot of the phases, capacitance over maximum capacitance ($C\ C_0^{-1}$ vs. *Frequency*) per each sample, real complex capacitance over maximum real complex capacitance ($C'\ C'_0{}^{-1}$ vs. *Frequency*) per each sample, imaginary complex capacitance over maximum imaginary complex capacitance ($C''\ C''_0{}^{-1}$ vs. *Frequency*) with relaxation time constants per each sample.

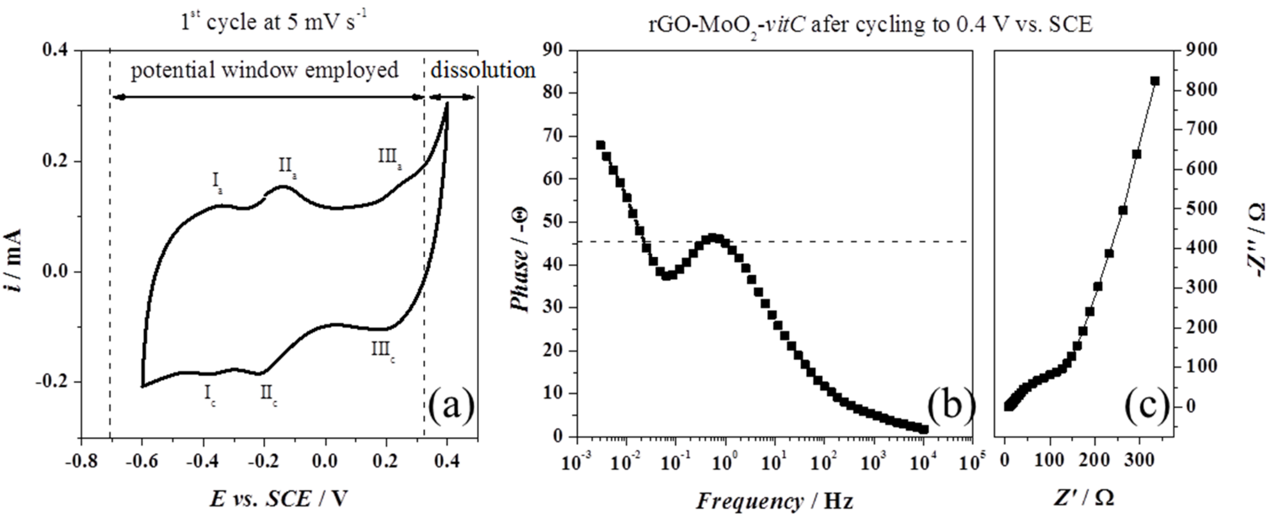

Figure S4. Experiments carried out 1 M Na_2SO_4: (a) Cyclic voltammetry up to 0.4 V, (b) Bode plot of the phase, and (c) Nyquist plot acquired at OCP after 100 cyclic voltammograms at 5 mV s^{-1} in the potential window of the experiment (a).

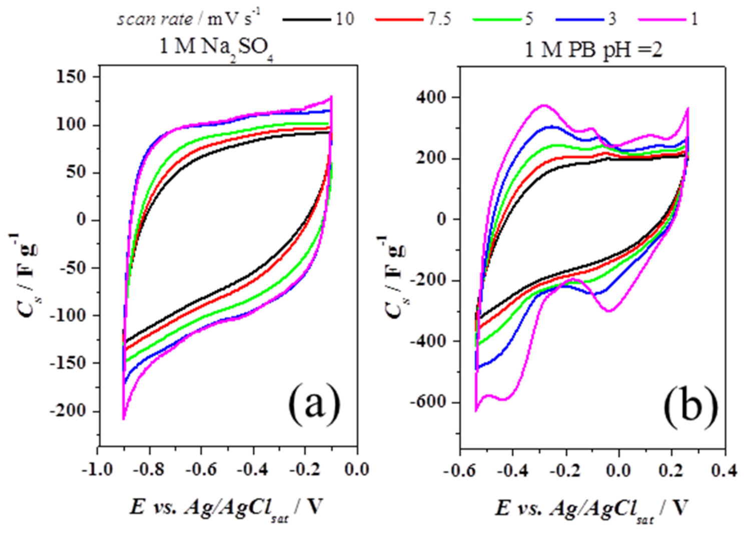

Figure S5. Differential voltammetry carried out in (a) 1 M Na_2SO_4 with the lower potential limit of -0.9 V and (b) in 1 M PB at pH = 2.

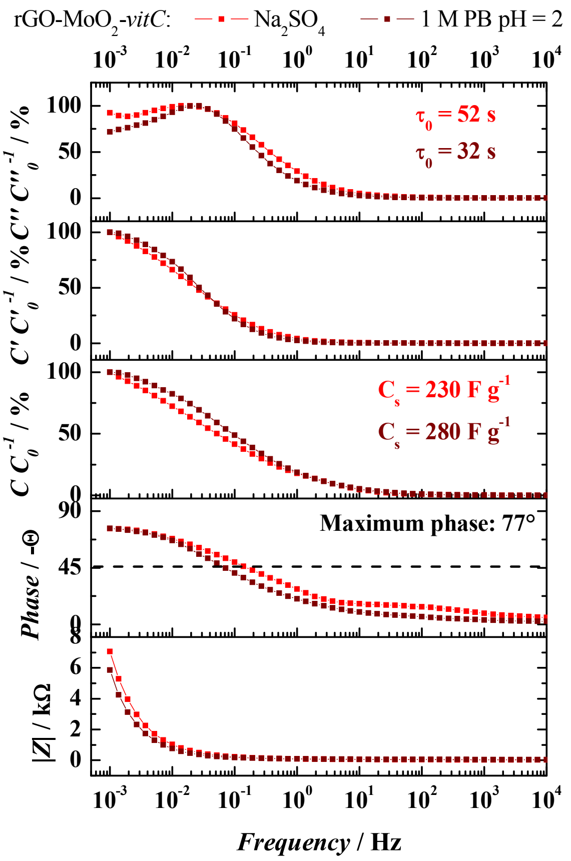

Figure S6. Comparison of rGO-MoO_2-*vitC* in 1 M Na_2SO_4 (red) and 1 M PB at pH = 2 (wine). From the bottom graph to the top: Bode plot of the modules, Bode plot of the phases, capacitance over maximum capacitance ($C\ C_0^{-1}$ vs. *Frequency*) per each sample, real complex capacitance over maximum real complex capacitance ($C'\ C'_0{}^{-1}$ vs. *Frequency*) per each sample, imaginary complex capacitance over maximum imaginary complex capacitance ($C''\ C''_0{}^{-1}$ vs. *Frequency*) with relaxation time constants per each sample.

- The equations employed for the estimation of the capacitance:

Calculation of Capacitance

Specific capacitance in three electrode cells was estimated as:

$$C_s = \frac{C}{m}$$

where C is the capacitance (F) and m is the mass of the electrode (g). In voltammetry, the capacitance is defined as the area under the current transient during one linear scan of the voltammogram normalized with respect to the potential window employed (ΔV), as:

$$C = \frac{\int_{t_1}^{t_2} i \, dt}{\Delta V}$$

while in charge-discharge cycles, the capacitance is estimated as the product of the current applied during the discharge cycle and the inverse of the slope of the chronopotentiogram, as:

$$C = i \frac{1}{\Delta V / \Delta t}$$

From AC impedance, the Cole-Cole plots were drawn according to:

$$C^{I}(\omega) = \frac{-Z^{II}(\omega)}{\omega |Z|^2}$$

$$C^{II}(\omega) = \frac{Z^{I}(\omega)}{\omega |Z|^2}$$

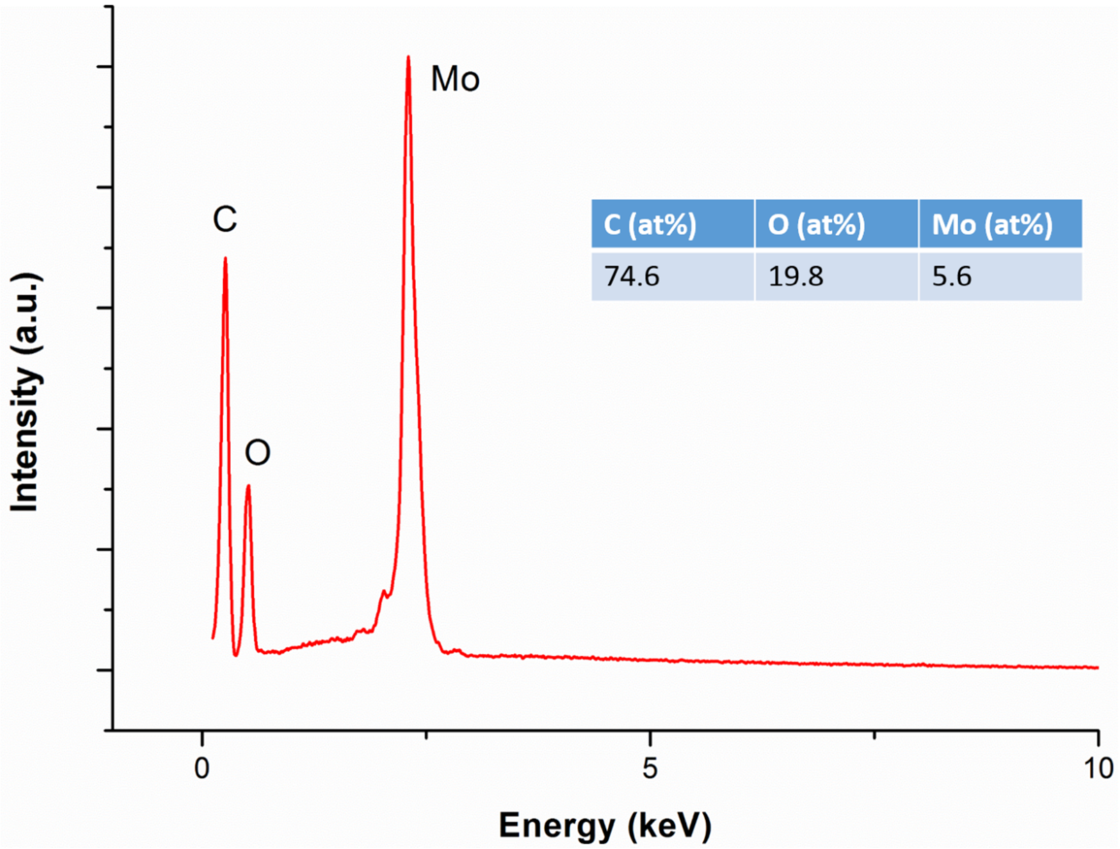

Figure S7. EDX spectrum of sample rGO-MoO2 with related semi-quantitative analysis.

© 2020 by the authors. Submitted for possible open access publication under the terms and conditions of the Creative Commons Attribution (CC BY) license (http://creativecommons.org/licenses/by/4.0/).
